# Effects of red pitaya juice supplementation on cardiovascular and hepatic changes in high-carbohydrate, high-fat diet-induced metabolic syndrome rats

**DOI:** 10.1186/1472-6882-14-189

**Published:** 2014-06-12

**Authors:** Nurul Shazini Ramli, Lindsay Brown, Patimah Ismail, Asmah Rahmat

**Affiliations:** 1 Department of Nutrition and Dietetics, Universiti Putra Malaysia, Serdang 43400 UPM, Malaysia; 2 School of Health, Nursing and Midwifery, University of Southern Queensland, Toowoomba QLD 4350, Australia; 3 Department of Biomedical Sciences, Universiti Putra Malaysia, Serdang 43400 UPM, Malaysia

**Keywords:** Red pitaya juice, Metabolic syndrome, High-carbohydrate high-fat diet

## Abstract

**Background:**

The fruit of *Hylocereus polyrhizus*, also known as red pitaya, and *buah naga* in Malay, is one of the tropical fruits of the cactus family, *Cactaceae.* Red pitaya has been shown to protect aorta from oxidative damage and improve lipid profiles in hypercholesterolemic rats probably due to phytochemicals content including phenolics and flavonoids. The aim of this study was to investigate the changes in cardiac stiffness, hepatic and renal function in high-carbohydrate, high-fat diet-induced obese rats following supplementation of red pitaya juice.

**Methods:**

Total 48 male Wistar rats were divided into 4 groups: corn-starch group (CS), corn-starch + red pitaya juice group (CRP), high-carbohydrate, high fat group (HCHF) and high-carbohydrate, high fat + red pitaya juice (HRP). The intervention with 5% red pitaya juice was started for 8 weeks after 8 weeks initiation of the diet. Heart function was determined *ex vivo* with Langendorff hearts while plasma liver enzymes, uric acid and urea were measured using commercial kits. Total fat mass was determined with Dual-energy X-ray absorptiometry (DXA) scan. Glucose uptake was measured with Oral Glucose Tolerance Test (OGTT). Liver and cardiac structures were defined by histology.

**Results:**

Supplementation of red pitaya juice for 8 weeks increased energy intake and abdominal circumference but no change in body fat and lean mass respectively. Also, there were a trend of uric acid and glucose normalization for HRP as compared to H-fed rats. Red pitaya juice treatment reduced ALP and ALT but caused significant increment in AST. Diastolic stiffness of the heart was reduced after supplementation of red pitaya juice in corn starch fed rats. However, the reduction was not significant in HRP rats in comparison with H rats.

**Conclusion:**

The present study concluded that red pitaya juice may serve as a complimentary therapy for attenuating some signs of metabolic syndrome.

## Background

Overweight and obesity are dramatically on the rise in recent decades. According to WHO [1], obesity contributed to double burden of diseases particularly diabetes (44%), ischemic heart diseases (23%), and certain types of cancer (7-41%). This is due to the metabolic abnormalities created by excessive fat accumulation like abnormalities of lipid in the blood, hypertension and impaired glucose tolerance, among which are the common features of metabolic syndrome [2]. In patients with metabolic syndrome, insulin resistance results in the impaired insulin activities in tissues like muscle, liver, kidney and fat leading to increase oxidative stress, pro-coagulant/anti-fibrinolytic and chronic pro-inflammatory state coupled with platelet hyper-aggregality [3]. Available evidences suggested the use of dietary intervention as an integral part of future approaches to prevent and treat obesity and its metabolic consequences [4]. Hence, this study focuses on cardiovascular and hepatic system as they are the ultimate consequences of obesity.

Diet-induced metabolic syndrome was found to be the closest model that at least shares the similar ethologic, and hence more representative of human pathophysiology of metabolic syndrome. Human consume high amount fat and carbohydrate like sucrose and fructose in their diet. Panchal et al. [5] studied the remodelling effect of high-carbohydrate high-fat diet-induced obesity in rats using condensed milk (39.5%), beef tallow (20%), and fructose (17.5%) with 25% fructose in drinking water and found that rats developed cardiovascular, metabolic, renal, hepatic and pancreatic changes. The complications includes obesity, increased fat accumulation in abdominal region, hypertension, insulin resistant, and impaired cardiac function, endothelial dysfunction as well as inflammation. Hence, it can be seen that a combination of high carbohydrate and high fat diet produce a more human-like model. This study only utilized male rats to avoid the influence of the oestrus cycle on food intake which may affect the diet-induced model [6].

Consumption of fruits and vegetables has long been linked to the prevention of oxidative stress related diseases like diabetes mellitus, cancer, heart disease, obesity and micronutrient deficiencies [7-10]. Eating fruits and vegetables can ensure the adequate supply of micronutrients, dietary fibers and phytochemicals which in turn maintain the body in a healthy state [11]. However, it is not clear which specific fruits and vegetables are most protective against certain diseases. Only few studies have examined the effect of specific fruits or juices on metabolic syndrome risk factors. A large prospective cohort study for 10.2 years on Swedish men and women found significantly inverse association of only apples, pears and green leafy vegetables with stroke [12].

Not all fruits are created equal particularly in terms of their phytonutrient contents which might influence their biological properties, and hence their efficacy in relation to specific diseases. The fruit of *Hylocereus polyrhizus*, also known as red pitaya, and *buah naga* in Malay, is one of the tropical fruits of the cactus family, *Cactaceae.* Polyphenols including flavonoids, betacyanins, vitamin C and fiber are among the main active constituents in red pitaya known to confer health benefit [13-15]. However, betacyanin fractions shown to display the highest reducing and radical scavenging capacities as compared to polyphenolic fractions [16]. Since the study on the physiological effects of red pitaya is still some distance from that of other fruits, it is interesting to investigate whether supplementation of 5% red pitaya juice can ameliorate the metabolic, hepatic and renal function in rats fed a high-carbohydrate, high-fat diet. To the best of our knowledge, this is the first study to evaluate the effect of red pitaya associated with hepatoprotection and cardioprotection.

## Methods

### Preparation of diet

Red pitaya was obtained from Queensland Australia. The identification of the fruit was done by a botanist from Biodiversity Unit, Institute of Biosciences, Universiti Putra Malaysia. The voucher number is SK-2440/14. The fruits were then cleaned, and the fruit pulp was squeezed using juice maker. Sample preparation was conducted in reduced light condition in order to minimize the pigment loss.

### Animals and diet

All experimental protocols were approved by the Animal Experimentation Ethics Committee of The University of Southern Queensland under the guidelines of the National Health and Medical Research Council of Australia. This study was a randomized control trial. The experimental groups consisted of 48 male Wistar rats (aged 8–9 weeks; weight 337 ± 5 g) supplied by and individually housed at The University of Southern Queensland animal house. All experimental groups were housed in a temperature-controlled, 12 hour light–dark cycle environment with *ad libitum* access to water and food. Daily body weight, feed and water measurements were taken to monitor the day-to-day health of the rats. The rats were randomly divided into six groups based on their diet: corn starch (C; n = 12); corn starch + red pitaya juice (CRP; 5% in the diet; n = 12); high-carbohydrate, high-fat (H; n = 12); High-carbohydrate, high-fat + red pitaya juice (HRP; n = 12). Fructose (25%) was added as drinking water for all high-carbohydrate, high-fat fed rats, while corn starch group was given normal water. The detailed macro- and micro-nutrient composition of the C and H diets are reported in our previous publications [17,18]. Red pitaya juice supplementation was administered for 8 weeks starting from 8 weeks after the initiation of the C or H diet.

### Oral glucose tolerance test

Basal blood glucose concentrations were measured in tail vein blood using a Medisense Precision Q.I.D glucose meter (Abbott Laboratories) after overnight (10–12 h) food deprivation. Fructose-supplemented drinking water in the H and HRP groups was replaced with normal water for the overnight food-deprivation period. The rats were given 2 g/kg body weight of glucose as a 40% solution via oral gavage. Blood samples from the tail vein were taken at 0, 30, 60, 90, and 120 minute following glucose administration.

### Body composition measurements

Body composition was measured on rats by dual-energy X-ray absorptiometry (DXA) using a Norland XR36 DXA instrument (Norland, Fort Atkinson, WI, USA) after 16 weeks of feeding and 2 days before terminal experiments. DXA scans were analysed using the manufacturer’s recommended software for use in laboratory animals (Small Subject Analysis Software, version 2.5.3/1.3.1; Norland Corp) [19]. The precision error of lean mass for replicate measurements, with repositioning, was 3.2%. Visceral adiposity index (%) was calculated as ([retroperitoneal fat (g) + omental fat (g) + epididymal fat (g)]/[body weight (g)]) × 100 and expressed as adiposity percent [17].

### Isolated heart preparation

Langendorf heart preparations were used to assess left ventricular function of the rats in all treatment groups. Terminal anaesthesia was induced via intraperitoneal injection of pentobarbitone sodium (Lethabarb, 100 mg/kg). Heparin (Sigma-Aldrich Australia) was administered (200 IU) through the right femoral vein and blood (~5 mL) was drawn out of the abdominal aorta. Isovolumetric ventricular function was measured by inserting a latex balloon catheter into the LV connected to a Capto SP844 MLT844 physiological pressure transducer and Chart software on a Maclab system (ADInstruments Australia and Pacific Islands). All left ventricular end-diastolic pressure values were measured while pacing the heart at 250 beats/min using an electrical stimulator. End-diastolic pressures were obtained starting from 0 mm Hg up to 30 mm Hg. The diastolic stiffness constant (k, dimensionless) was calculated as in previous studies [20]. +dP/dt and - dP/dt were calculated as the mean rate of contraction and relaxation, respectively, of at least 50 beats with the heart paced at 250 beats/min, and the end-diastolic pressure was maintained at approximately 10 mmHg.

### Organ weights

The right ventricle and LV were separated after perfusion experiments and weighed. Liver and abdominal fat were immediately removed at the time of the heart removals for perfusion experiments and blotted dry for weighing. Perirenal, epididymal, and omental fat were together weighed as abdominal fat. Organ weights were normalized relative to the tibial length at the time of their removal (in mg/mm).

### Plasma biochemistry analysis

Blood was collected into heparinized tubes and then centrifuged at 5,000 *g* for 15 minutes. Plasma samples were separated and into Eppendorf tubes and stored at -80°C for analysis. Enzymatic activities and analyte concentrations in the plasma (AST, ALP, ALT) were determined using kits and controls supplied by Olympus using an Olympus analyzer (AU 400). Plasma glucose, uric acid and urea were estimated using a commercial kit according to the manufacturer-provided standards and protocol using a Roche/Hitachi cobas c system.

### Histology

The liver and heart tissues for 2 rats (n = 2) from each group were exclusively taken for histopathological analysis. The samples were immediately fixed in 10% formalin for 3 days to remove the traces of blood from the tissue. After that, the samples were dehydrated, embedded in paraffin wax and then cut into thin sections (5–6 μm). In order to determine the inflammatory cell infiltration, the liver and heart tissue sections were stained with hematoxylin and eosin. Picrosirius red staining was used to study collagen deposition in left ventricle of the heart and was analysed using laser confocal microscopy (Zeiss LSM 510 upright confocal microscope). From each tissue sample, three slides were prepared and two random, nonoverlapping fields were selected from each slide. A representative picture was randomly selected from each group.

### Statistical analysis

All data were presented as mean ± SEM. A total of 4 groups were analysed using two-way analysis of variance (ANOVA). Each group consists of 12 rats. All group data were tested for variance using Bartlett’s test. Variables that were not normally distributed were transformed (using log 10 function) prior to statistical analysis. The effects of diet, treatment and their interactions were tested by two-way analysis of variance. When interaction and/or the main effects were significant, means were compared using Newman-Keuls multiple-comparison post hoc test. Where transformations did not result in normality or constant variance, a Kruskal-Wallis nonparametric test was performed. P <0.05 was considered significant. All statistical analyses were performed using GraphPad Prism version 5.00 for Windows (San Diego, CA, USA).

## Results

### Dietary intake

The effects of red pitaya juice on food intake, water intake and energy intake were determined after 8 weeks of supplementation. Table 1 shows red pitaya supplementation significantly increase food intake only in CRP group. Average food intake for corn starch fed control group (C) was 36.33 ± 0.9 g/day increased to 40.40 ± 0.7 g/day for CRP group. On the other hand, no change in food intake was observed in high-carbohydrate, high-fat diet supplemented with red pitaya (27.29 ± 1.3 and 27.74 ± 2.6 g/day for H and HRP groups, respectively). As shown in Table 1, red pitaya supplementation did not change water intake in either group (29.57 ± 2.6, 30.88 ± 1.8, 30.15 ± 1.6, 26.47 ± 2.3, 29.56 ± 5.0 and 21.75 ± 1.1 ml/day for C, CRP, H, and HRP groups, respectively). Even though food and water intake were similar in high-carbohydrate, high-fat diet feeding throughout the study period, rats on this diet supplemented with red pitaya juice had increased in energy intake (512.7 ± 8.3 and 655.2 ± 28.7 kJ/day for both CRP and HRP) (Table 1).

**Table 1:** Dietary intakes, body composition, organ wet weights and plasma biochemistry analysis in C, CRP, H and HRP diet-fed rats

**Variable**	**C**	**CRP**	**H**	**HRP**	***P*-value**		
					**Diet**	**Treatment**	**Interaction**
Food intake, g/d (n = 7-8)	36.3 ± 0.9^b^	40.4 ± 0.7^a^	27.3 ± 1.3^c^	27.7 ± 2.6^c^	<0.0001	0.0375	0.07
Water intake, ml/d (n = 7-8)	29.6 ± 2.6	30.9 ± 1.7	26.5 ± 2.3	29.6 ± 5.0	0.06	0.3	0.41
Energy intake, kJ/d (n = 8)	405.5 ± 10.2^d^	512.7 ± 8.3^c^	569.2 ± 28.7^b^	655.2 ± 28.7^a^	0.8041	<0.0001	<0.0001
Body weight gain (8–16 weeks),% (n = 8)	7.1 ± 1.1^bc^	11.6 ± 2.6^b^	19.1 ± 1.4^a^	19.7 ± 1.5^a^	<0.0001	0.12	0.19
Total body fat mass, g (n = 8)	77.5 ± 5.8^b^	110.6 ± 10.4^b^	210.8 ± 27.6^a^	230.4 ± 23.1^a^	<0.0001	0.23	0.85
Total body lean mass, g (n = 8)	296.9 ± 10.6	305.2 ± 3.0	296.8 ± 15.4	286.2 ± 16.0	0.67	0.42	0.31
Abdominal circumference, cm (n = 8)	18.9 ± 0.2^d^	20.2 ± 0.3^c^	22.1 ± 0.4^b^	24.7 ± 0.5^a^	<0.0001	<0.0001	0.0403
**Tissue wet weights, mg/mm tibial length (n = 8)**							
Liver (n = 7-8)	248.8 ± 12.0^b^	258.0 ± 16.4^b^	332.5 ± 1.0^a^	363.2 ± 8.6^a^	<0.0001	0.18	0.41
Kidneys (n = 7-8)	47.92 ± 1.3^b^	54.62 ± 1.7^b^	57.31 ± 3.1^a^	58.62 ± 1.5^a^	0.0299	0.0856	< 0.0001
Spleen (n = 7-8)	17.27 ± 1.1	19.53 ± 1.2	19.1 ± 0.3	16.11 ± 1.6	0.0042	0.9573	0.1704
**Plasma biochemistry analysis**							
Urea (mmol/L) (n = 7-8)	2.74 ± 0.62	2.94 ± 0.31	2.90 ± 0.33	2.11 ± 0.12	0.1463	0.6032	0.5296
uric acid (μmol/L) (n = 7-8)	32.6 ± 5.36^b^	45.4 ± 1.37^a^	26.6 ± 2.2^bc^	16.4 ± 2.36^c^	0.0014	0.0793	< 0.0001
Glucose, mmol/L (n = 5-8)	11.2 ± 0.61^ab^	12.6 ± 1.19^a^	12.4 ± 0.45^ab^	9.7 ± 0.44^b^	0.0118	0.4315	0.6681

Each value is a mean ± S.E.M. Number of repetitive experiments indicated within parenthesis. Means within a row with unlike superscripts letters a, b, c, d differ significantly (p < 0.05). C, corn starch diet; CRP, corn starch + red pitaya juice; H, high fat diet; HRP, high fat diet + red pitaya juice.

### Body weight

**Figure 1:**
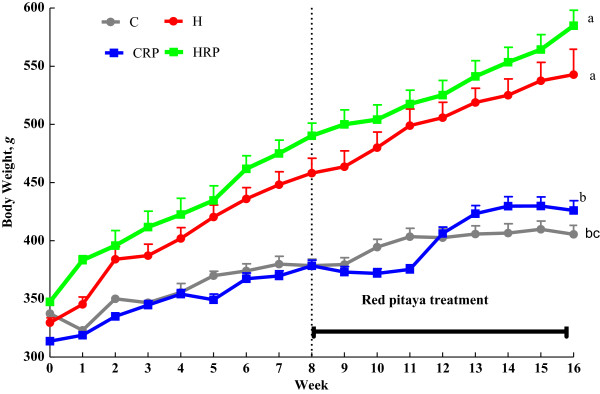
**Body weight of obese rats supplemented with red pitaya juice for 8 weeks.** Data are presented as mean ± SEM, n = 5–8. Values marked with different letters are significantly different at the level of p < 0.05. C, corn starch diet; CRP, corn starch + red pitaya juice; H, high fat diet; HRP, high fat diet + red pitaya juice.

Figure 1 displays body weights of obese rats supplemented with red pitaya juice. Initially, there was no difference in body weight for all the treatment groups. After 8 weeks initiation of the diet, the increased in body weights in high-carbohydrate, high-fat diet-fed rats were significantly (p < 0.05) greater than corn starch diet–fed rats. At the end of 8 weeks, supplementation of red pitaya juice failed to prevent further weight gain compared with C and H diet-fed rats (Figure 1). Consequently, both supplemented rats showed the trend of increasing body weight although the difference was not significant (p < 0.05) (Figure 1).

### Plasma biochemistry analysis

The wet weight of the liver and kidneys were significantly greater (p < 0.05) in the high carbohydrate, high-fat diet-fed rats (H) compared with corn starch fed-rats (C) (Table 1). However, red pitaya did not produce any significant changes to liver and kidneys wet weight. Contrarily, there were no difference in wet weight of the heart and spleen for all the groups (C, CRP, H and HRP). No changes in plasma urea concentration in red pitaya supplemented rats compared with C-fed and H-fed rats (Table 1). Furthermore, red pitaya supplementation showed the trend of uric acid and glucose normalization for HRP as compared to H-fed rats (Table 1). Conversely, plasma uric acid concentration was increased significantly in CRP rats (p < 0.05) compared with C-fed rats (Table 1).

### Glucose tolerance and hepatic function

**Figure 2:**
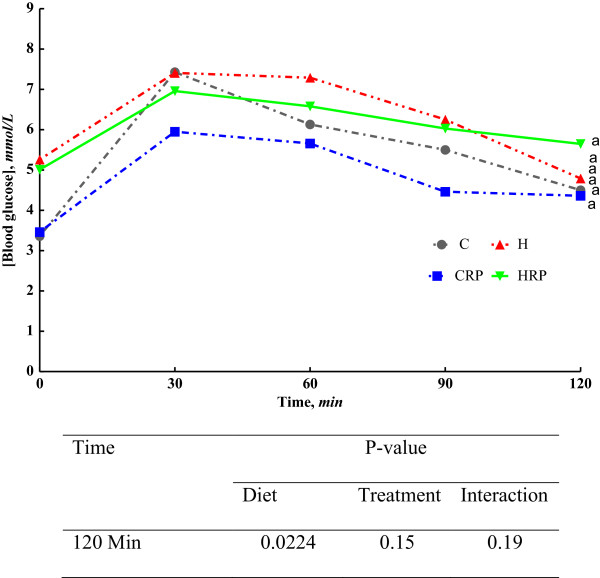
**Oral Glucose Tolerance Test (OGTT) at 16 weeks from C, CRP, H and HRP rats.** Data are presented as mean ± SEM, n = 5–8. End-point means without a common alphabet in each data set significantly differ at p < 0.05. C, corn starch diet; CRP, corn starch + red pitaya juice; H, high fat diet; HRP, high fat diet + red pitaya juice.

**Figure 3:**
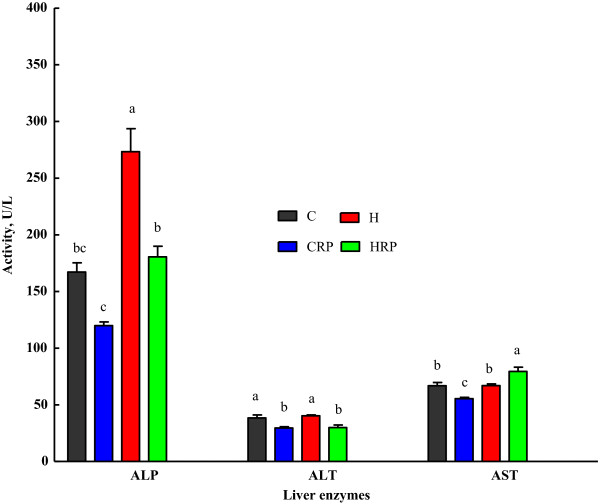
**Plasma concentrations of liver enzymes of obese rats supplemented with red pitaya juice for 8 weeks.** Data are presented as mean ± SEM, n = 5–8. Values marked with different letters are significantly different at the level of p < 0.05. ALP, alkaline phosphatase; ALT, alanine transaminase; AST, aspartate transaminase. C, corn starch diet; CRP, corn starch + red pitaya juice; H, high fat diet; HRP, high fat diet + red pitaya juice.

Red pitaya supplemented groups showed impaired glucose tolerance even after 8 weeks supplementation (Figure 2). Overnight fasting blood glucose differs significantly between H fed rats and C fed rats. Oral administration of 2 g/kg body weight of glucose resulted in increased blood glucose concentration at 30 minutes and 60 minutes for C, CRP, H and HRP respectively. Then, blood glucose was slowly cleared from the blood in H fed rats as observed at 90 minutes. At the end of 120 min, HRP rats failed to reduce blood glucose concentration to the basal value. The similar results were observed for CRP rats as compared to C-fed rats (Figure 2). Insulin concentration in all the rats were unable to be detected using cobas c system (data was not shown). High-carbohydrate, high-fat feeding resulted in elevated levels of plasma alanine transaminase (ALT), aspartate transaminase (AST) and alkaline phosphatase (ALP) as the markers of liver function (Figure 3). HRP diet feeding decreased the ALP and ALT activities but significantly increased (p < 0.05) AST activity (Figure 3). Plasma ALP activity was not affected in CRP group while plasma ALT was significantly decreased in CRP compared with C-fed rats (Figure 3). However, plasma AST activity was decreased in CRP group (p <0.05) (Figure 3).

### Cardiovascular function

**Figure 4:**
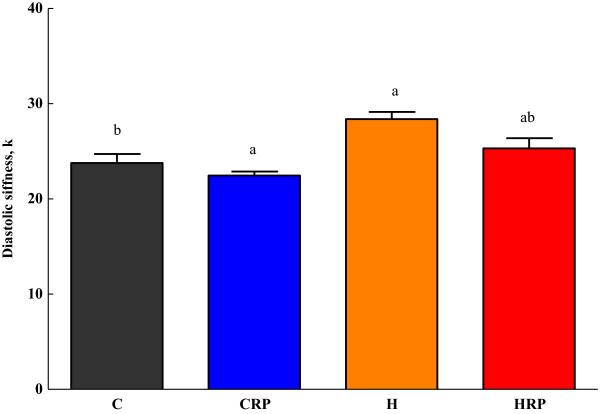
**Diastolic stiffness of obese rats supplemented with red pitaya juice for 8 weeks.** Data are presented as mean ± SEM, n = 5–8. Values marked with different letters are significantly different at the level of p < 0.05. C, corn starch diet; CRP, corn starch + red pitaya juice; H, high fat diet; HRP, high fat diet + red pitaya juice.

The measurement of *ex-vivo* cardiac function of the obese rats was accomplished by conducting Langendorff isolated heart. Figure 4 demonstrates that high carbohydrate, high fat diet-fed rats showed marked increased in diastolic stiffness in comparison with corn starch diet-fed rats (Figure 4). Results revealed that diastolic stiffness of the heart was reduced after supplementation of red pitaya juice in corn starch fed rats (Figure 4). However, the reduction was not significant in HRP rats in comparison with H rats (Figure 4).

### Liver and cardiovascular structure

**Figure 5:**
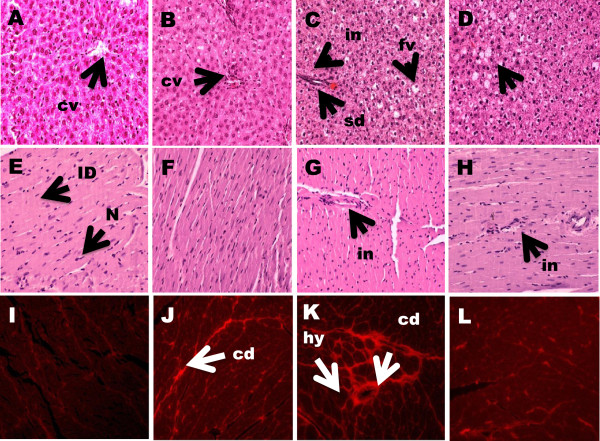
**Histopathology of liver and heart of obese rats supplemented with red pitaya juice for 8 weeks. (A-D)** Haematoxylin and eosin staining of liver section showing hepatocytes with enlarged fat vacuoles (marked as “fv”) (× 20), sinusoids dilatation (marked as “sd”) and inflammatory cells infiltration around the sinusoids (marked as “in”) (× 20). **(E-H)** Hematoxylin and eosin staining of left ventricle showing inflammatory cell infiltration (× 20) (marked as “in”) as dark spots outside the myocytes. **(I-L)** Picrosirius red staining of left ventricle showing interstitial collagen deposition (× 20) (marked as “cd”) and hypertrophied cardiomyocytes (marked as “hy”). **A,E,I**, corn starch diet; **B,F,J**, corn starch + red pitaya juice; **C,G,K**, high fat diet; **D,H,L**, high fat diet + red pitaya juice.

Histological evaluation of liver tissues showed negligible fat vacuoles accumulation without inflammatory cells in C group (Figure 5A). After eight weeks supplementation, CRP (Figure 5B) displayed less fat vacuoles accumulation but demonstrated marked hepatocyte ballooning with cornered nucleus. High carbohydrate, high fat feeding for 16 weeks resulted in augmented accumulation of fat vacuoles in the hepatocytes with sinusoids dilatation and increased inflammatory cell infiltration in H group (Figure 5C). Red pitaya supplementation reduces the fat vacuoles and infiltration of inflammatory cells (Figure 5D).Histological evaluation of left ventricle tissues after fed with high carbohydrate, high fat diet for 8 weeks resulted in greater inflammatory cells infiltration (Figure 5G) compared to CS rats (Figure 5E). Similarly, H rats showed higher collagen deposition and hypertrophied cardiomyocytes (Figure 5K) than CS rats (Figure 5I). After supplementation with red pitaya for 8 weeks, HRP rats showed slightly greater infiltration by inflammatory cells into the left ventricle (Figure 5H). However, HRP rats showed reduced collagen deposition (Figure 5L). On the other hand, no changes in inflammatory cell infiltration were observed in CRP rats (Figure 5F) while collagen deposition was greater in CRP rats (Figure 5J) compared to CS rats (Figure 5I).

## Discussion

In this study, red pitaya juice was used as an intervention in order to investigate the ability of the fruit juice to reverse the abnormalities in body composition, metabolic, liver, kidneys and heart function in high carbohydrate, high-fat diet induced obese rats as a model of metabolic syndrome. Previous research confirmed that rats fed with high carbohydrate and high fat diet for 8 weeks produced abdominal obesity, impaired glucose tolerance, hypertension, dyslipidemia, inflammation, endothelial dysfunction, cardiac fibrosis together with increased stiffness of the heart [17,18]. All these changes closely mimic the human metabolic syndrome.

Results revealed that red pitaya supplementation increased food intake only in corn starch diet-fed control rats while no changes were observed in red pitaya juice supplementation for high carbohydrate, high-fat diet-induced obese rats and the corn starch diet fed control rats. In other words, the intervention does not affect satiety of obese rats as the quantity of food taken was shown to be regulated by the factors involved in satiety perception [21]. There was report suggesting that the food intake was influenced by the sensitivity of the rats to the palatability of the food in the diet [22]. Therefore, it was proposed that the lean control rats in the present study were more sensitive to the palatability of red pitaya juice as compared to high carbohydrate, high-fat diet-induced obese rats. Food rich in sugar and fat is an example of palatable foods that inhibit the satiety signals and up regulate hunger sensation [23]. Despite of that, energy intake was increased significantly for red pitaya supplemented rats suggested that red pitaya contains higher amount of energy-supplying macronutrients. Red pitaya added up to the total energy content of the diet, leading to progressive increment in body weight, and total body fat throughout the intervention period. A growing body evidences showed that eating fruits can reduce energy density and reduce energy density is associated with lower energy intake and decrease body weight [24]. Furthermore, Flood-Obbagy and Rolls [25] indicated that consumption of fruits before meal can increase satiety and reduce energy intake but the effects are different for different forms of fruits. Whole fruit is related to more chewing while fruit juices do not require chewing at all. The amount of chewing is then linked to cephalic-phase responses that control the food intake [26,27]. This may explain the reduce risk of type 2 diabetes mellitus following consumption of specific whole fruits particularly grapes, apples, bananas, and blueberries in prospective longitudinal cohort study among women and men from Nurses’ Health study and Health Professionals Health Study respectively [28].

It is fascinating to address the suppression effects of red pitaya on body fat for HRP rats. As the diet was continue for 8 weeks, supplementation of red pitaya did not result in further weight gain for obese rats. A current review on regulation of body fat mass in obesity indicated that body resisted fat loss once obesity is established [21] probably through adaptive mechanism of increasing metabolic rate. Contradictory, total body fat mass in corn starch fed rats were significantly elevated when supplemented with red pitaya. It could be the normal physiological responses upon increasing energy intake in red pitaya supplemented rats. In conjunction with that abdominal circumference were increased in CRP and HRP rats. A possible explanation for this might be that the increase in fat mass in red pitaya fed rats was deposited in abdominal region, hence it can be detrimental to their health. It was expected to see no changes in lean mass for red pitaya supplemented rats as lean mass is a metabolically active tissues required for maintaining basic cell function, thus was not affected by treatment.

Liver and kidneys from H fed rats had higher wet weights as compared to C fed rats as observed in previous study [17] while no changes in wet weight of spleen and hearts. Likewise, a recent study presented liver abnormalities comprises of higher liver weight, increases in alanine transaminase (ALT) and aspartate transaminase (AST) and the occurrence of inflammatory infiltrates in rats while renal abnormalities were evidenced from the increased in weight, fat accumulation, and glomerular sclerosis with consumption of high fat diet [29]. A probably explanation for this defects is through the deposition of lipid in the liver upon fructose exposure which in turn conquer most of the hepatocytes volume and upset the liver structure and function [30]. Besides, some glucose transporter like GLUT2 may be involved in the transport of fructose to the kidney [31]. Besides, red pitaya-fed rats showed a trend of reduction in stiffness of the heart. Previous studies reported that diastolic stiffness, cardiac fibrosis, and elevated superoxide production were directly associated with each other [18]. Therefore, it could be that the accumulation of interstitial cardiac collagen was reduced [32] following red pitaya supplementation, hence showed the trend of ameliorating cardiac stiffness.

To have better insight of liver function, the enzymes activities were measured. The collective measurement of alkaline phosphatase (ALP), alanine transaminase (ALT) and aspartate transaminase (AST) reflects the severity of liver damage. ALP is induction enzyme that is mainly found in the cell membrane of the liver and the elevation of this enzyme indicates primary hepatic disease like cholestatic liver disease. Although the increase in ALP can be result from extra-hepatic sources like bone marrow and skeletal muscle, the effects was minimal. Whilst, both ALT and AST are leakage enzymes, and their elevation indicate significant hepatocellular damage. The possible causes may vary including toxicity, inflammation, hypoxia and tissue trauma [33]. However, AST is also found in heart, kidneys, brain, and skeletal muscle and had been used as a nonspecific marker for myocardiac infarction [34]. Results revealed that red pitaya juice treatment significantly reduced ALP and ALT but caused significant increment in AST. Therefore, the present results suggest that red pitaya juice ameliorated liver damage due to high carbohydrate, high fat feeding. The reduction of liver enzymes after the intervention probably indicated the decrease in deposition of fat and the degree of necrosis in liver cells [35]. In contrast, the elevated level of AST could be due to myocardial damage as the heart muscle is rich in aminotransferase enzymes especially AST. The decrease in ALT has been shown to have protective effects from death diabetes and Ischemic heart disease in adults from National Health and Nutrition Examination Survey (NHANES) III [36]. On the other hand, the increased in ALT has been associated with systemic and hepatic insulin resistance, along with increase in the secretion of insulin and reduces insulin clearance from the liver [37]. Hence, the present study showed that red pitaya juice is an important mediator for attenuation of liver injury. Histopathological evidence of liver and heart section after supplementation with 5% red pitaya juice revealed mild improvement in hepatic vacuolation and no inflammation was seen during microscopic examination. Also, the collagen deposition was reduced in left ventricle of the heart. This results was in line with the reduce ALP and ALT enzymes indicative of improvement in liver injury as well as consistent with a slightly reduction in diastolic stiffness. Previous report indicated that intervention with 5% purple carrot juice was also able to reverse the structural damage in the liver caused by high fat feeding by showing minimal signs of microvesicular steatosis [18].

## Conclusion

In summary, the present data provide scientific evidences that red pitaya juice may provide protection against liver damage and may reduce the stiffness of the heart. Red pitaya juice contained multiple bioactive compounds which might act synergistically to produce the protective effects. However, the effects of the specific constituents are not investigated in the present study. Therefore, it is suggested to conduct future study in order to elucidate the possible active compounds responsible for the effects. Besides, further study with higher doses of red pitaya in diet-induced obesity model can be conducted in future to examine the ability of this fruit for the treatment of obesity related diseases. It is also crucial to take into consideration the timing of fruit juice consumption. This is because eating fruit before meals may reduce energy intake by means of increasing fiber intake. The therapeutic effects of specific fruits should be investigated rather giving general recommendation to the general population.

## Competing interests

The authors declare that they have no competing interests.

## Authors’ contribution

NSR: preparation of the draft, contribution to conception, design, acquisition of data, analysis and interpretation of data. LB: contribution to conception, design of the study and interpretation of data PI: contribution to the interpretation of data. AR: data analysis and final approval of the version for publishing. All authors read and approved the manuscript.

## Pre-publication history

The pre-publication history for this paper can be accessed here:

http://www.biomedcentral.com/1472-6882/14/189/prepub
